# Clinical activity of regorafenib in *PDGFRA*-mutated gastrointestinal stromal tumor

**DOI:** 10.4155/fso.15.33

**Published:** 2015-11-01

**Authors:** Thomas Grellety, Michèle Kind, Jean-Michel Coindre, Antoine Italiano

**Affiliations:** 1 Department of Medical Oncology, Institut Bergonié, Bordeaux, France; 2 Department of Radiology, Institut Bergonié, Bordeaux, France; 3 Department of Pathology, Institut Bergonié, Bordeaux, France

**Keywords:** GIST, *PDGFRA* mutation, regorafenib

## Abstract

Gastrointestinal stromal tumor (GIST) is the most frequent mesenchymal tumor of the gastrointestinal tract and one of the most frequent sarcoma. Mutually exclusive *KIT* and *PDGFRA* mutations are central events in GIST pathogenesis, and their understanding is crucial because specific treatment targeting oncogenic KIT and PDGFRA activation (especially imatinib) has become available. The most frequent *PDGFRA* mutation (D842V) is associated with primary resistance to imatinib. Data related to regorafenib efficacy in *PDGFRA*-mutated GIST are lacking. We report here a case report of a prolonged response with regorafenib in a patient with a *PDGFRA-*mutated GIST.

## Abstract (lay abstract)

This report describes the clinical efficacy of regorafenib in a patient with metastatic gastrointestinal stromal tumor bearing a mutation of the *PDGFRA* exon 18 gene.

**Figure 1.**
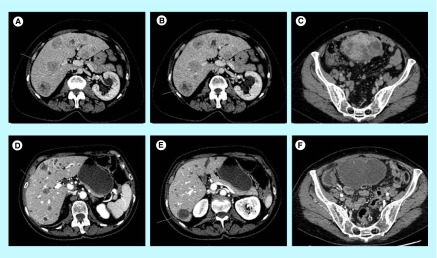
Response to regorafenib of a *PDGFRA* mutated GIST. Response to regorafenib in a patient with liver metastasis **(A, B, C)** before treatment; **(D, E, F)** response maintained 18 months after treatment onset) from a *PDGFRA* exon 18 mutated gastrointestinal stromal tumor.

## Case report

In December 2012, a 69-year-old woman presented with abdominal discomfort. CT scan revealed an 11 cm long pelvic mass, probably developed from the small intestine with diffuse liver metastasis. There was no remarkable past medical history. Histological examination of the mass biopsy allowed the diagnosis of malignant gastrointestinal stromal tumor (GIST). Genotyping showed a *PDGFRA* mutation (p.Asp842_Asp846delinsGlu) in exon 18. She started treatment with an investigational KIT/PDGFRA tyrosine kinase inhibitor (TKI) in March 2013. The first tumor evaluation performed 6 weeks after treatment onset showed unequivocal tumor progression according to CHOI and RECIST criteria consonant with an increase of functional symptoms. Imatinib primary resistance coupled with data available on sunitinib resistance to *PDGFRA-D842V* mutation led to offer the patient to start second-line regorafenib in April 2013 in the context of a compassionate program. The main side effects were grade II hypertension, grade II asthenia, grade II hypothyroidism, grade III diarrhea (NCI CTCAE v3), required dose reduction at 120 mg per day 4 months after treatment onset and improved within 4 weeks. Clinical improvement appears within the first month of treatment and tumor evaluation showed partial response according to CHOI and stable disease according RECIST 1.1 criteria, respectively. Twenty months after treatment onset, the patient is still under treatment and partial response maintained (Figure 1).

## Discussion & conclusion

GIST is the most common nonepithelial cancer developed in the gastrointestinal tract and principally develops on the stomach or small bowel and is one of the most common soft tissue sarcoma [1]. It remains a rare disease with an annual incidence rate of 0.68/100,000 persons in USA [2]. Most GISTs are characterized by oncogenic mutations in either *KIT* or *PDGFRA*, which probably play a central role in tumor initiation. *KIT* activating mutations account for 75–80% of all GISTs and allow targeted treatment with different TKIs such as imatinib or sunitinib [3]. TKI use improves the median overall survival to around 5 years, about five times more than before TKI use [4]. *PDGFRA* is mutated in 5–10% of GISTs principally in the exon 12, 14 and 18 (juxtamembrane domain, first tyrosine kinase domain and activation loop, respectively) [3,5]. The *PDGFRA* D842V (exon 18) mutation accounts for 60% of all *PDGFRA* mutations known in GISTs [3,5]. This mutation as well as the DI842–843IM and the RD841–842KI mutations confer primary resistance to imatinib [6] and sunitinib [7] whereas the sensitivity of other ones is maintained or unknown [6].

Regorafenib is a multikinase inhibitor that blocks several tyrosine kinase receptors such as KIT, vascular endothelial growth factor receptors, RET, BRAF and platelet-derived growth factor receptors. The antitumor effect of regorafenib in GIST may result from a direct inhibition of KIT and *PDGFRA* but also from an inhibition of other pathways such as Raf/ERK/MEK signaling [8]. Regorafenib showed promising results in a single-arm Phase II trial [8] and a significant improvement of progression-free survival in comparison to placebo in a Phase III trial including patients with advanced (GIST) refractory to imatinib and sunitinib [8,9]. Regorafenib has been approved for patients with advanced GIST who progressed on those two prior treatments. Regorafenib is well tolerated and toxicity principally lies on hypertension, hand-foot skin reaction and diarrhea [8,9]. Currently, alternative treatments could be off-label use of another KIT/PDGFRA specific TKI or clinical trial inclusion.

Given the rarity of metastatic GIST with *PDGFRA* mutation (11% in a large cohort) [10], there are no data related to the efficacy of regorafenib in this specific molecular subtype. Indeed, no patients with *PDGFRA* mutation were included in the Phase II study and no data were provided about the efficacy of regorafenib according to the primary mutational status in the Phase III trial. The mutation reported here was rare but previously described [6]. There was no preclinical data related to its biological role and to its sensitivity to imatinib. Our report represents the first published clinical evidence of the potential efficacy of regorafenib in patients with exon 18 *PDGFRA* mutations. Moreover, the observed response is prolonged as it can be seen with imatinib or sunitinib.

## Future perspective

Significant disease control is obtained by targeting RTK-driven GISTs (KIT and *PDGFRA*), thanks to molecular screening for those RTK gene mutations. Imatinib is indicated for the firs-line treatment of adult patients with KIT (CD117)-positive unresectable and/or metastatic malignant GIST. However, the majority of PDGFRA mutations are associated with primary resistance to imatinib. Given the low incidence of *PDGFRA* mutation in GIST and the low rate of metastatic relapse associated with this mutational pattern, specific clinical data about the management of patients with advanced *PDGFRA*-mutated GIST are almost nonexistent. Robust preclinical data assessment for TKI efficacy according to specific mutation of rare pro-oncogenic RTK such as *PDGFRA* should be encouraged. This will lead to achieving a real personalized and adapted care for each specific GIST. This rationale also needs to be encouraged for secondary resistant mutation in order to counteract resistance.

**Executive summary**

Gastrointestinal stromal tumor (GIST) is the most frequent mesenchymal tumor of the gastrointestinal tract and one of the most frequent sarcoma.
Approximately 80% of all GISTs contain a mutation in the KIT receptor tyrosine kinase that results in constitutive activation of the protein. The availability of specific, molecular-targeted therapy with KIT/PDGFRA tyrosine kinase inhibitors such as imatinib has dramatically improved the outcome of patients with GIST.
Approximately 5–8% of GISTs harbor a mutation in *PDGFRA. PDGFRA*-mutant GIST may differ from *KIT*-mutant GIST particularly in terms of sensitivity to tyrosine kinase inhibitors.
We report the first case of response to regorafenib in a patient harboring a *PDGFRA*-mutated GIST.
